# Heterogeneous-Nucleation Biosensor for Long-Term Collection and Mask-Based Self-Detection of SARS-CoV-2

**DOI:** 10.3390/bios13090858

**Published:** 2023-08-30

**Authors:** Yi Su, Sumin Bian, Dingyi Pan, Yankun Xu, Guoguang Rong, Hongyong Zhang, Mohamad Sawan

**Affiliations:** 1 College of Biomedical Engineering & Instrument Science, Zhejiang University, Hangzhou 310013, China; suyi@westlake.edu.cn (Y.S.); dpan@zju.edu.cn (D.P.); 2 CenBRAIN Neurotech, School of Engineering, Westlake University, Hangzhou 310030, China; biansumin@westlake.edu.cn (S.B.); xuyankun@westlake.edu.cn (Y.X.); rongguoguang@westlake.edu.cn (G.R.); zhanghongyong@westlake.edu.cn (H.Z.)

**Keywords:** biosensors, Au NCs, SARS-CoV-2, COVID-19, mask, heterogeneous nucleation

## Abstract

The effective control of infectious diseases, including Severe Acute Respiratory Syndrome Coronavirus 2 (SARS-CoV-2) infection, depends on the availability of rapid and accurate monitoring techniques. However, conventional SARS-CoV-2 detection technologies do not support continuous self-detection and may lead to cross-infection when utilized in medical institutions. In this study, we introduce a prototype of a mask biosensor designed for the long-term collection and self-detection of SARS-CoV-2. The biosensor utilizes the average resonance Rayleigh scattering intensity of Au nanocluster-aptamers. The inter-mask surface serves as a medium for the long-term collection and concentration enhancement of SARS-CoV-2, while the heterogeneous-nucleation nanoclusters (NCs) contribute to the exceptional stability of Au NCs for up to 48 h, facilitated by the adhesion of Ti NCs. Additionally, the biosensors based on Au NC-aptamers exhibited high sensitivity for up to 1 h. Moreover, through the implementation of a support vector machine classifier, a significant number of point signals can be collected and differentiated, leading to improved biosensor accuracy. These biosensors offer a complementary wearable device-based method for diagnosing SARS-CoV-2, with a limit of detection of 10^3^ copies. Given their flexibility, the proposed biosensors possess tremendous potential for the continuous collection and sensitive self-detection of SARS-CoV-2 variants and other infectious pathogens.

## 1. Introduction

Infectious diseases cause serious health and social problems [1], as is evident from the pandemic caused by the Severe Acute Respiratory Syndrome Coronavirus 2 (SARS-CoV-2). To date, various state-of-the-art technologies have been established for the precise detection of SARS-CoV-2 [2,3], including the golden standard quantitative reverse transcription-polymerase chain reaction (RT-PCR) [4] for viruses, serological tests for determining a previous infection, computed tomography or nuclear magnetic resonance methods for diagnosis at hospitals [5], and antigen-based biosensing for spike proteins or viruses [6]. Despite their multiple advantages, these technologies are still far from being desirable. They require professional institutions to measure one single sample per individual and do not allow for continuous collection and monitoring. Moreover, the entire detection process, from sampling to pre-treatment, analysis, and data reporting, typically takes several hours, resulting in delayed decision-making for treatment. A technique that would allow for long-time collection and self-detection, and that could deliver quick results, preferably directly to the people who are wearing facemasks every day during the pandemic, would benefit both the public as well as the healthcare systems.

A mask-based biosensor would be one of the potential tools for reaching the above-mentioned goals by detecting SARS-CoV-2 in our breath. The latest clinical studies have demonstrated that the breath emission rate of SARS-CoV-2 from infected patients [7] reaches an output of from one thousand to one hundred thousand copies [8] per min in a facemask [9]. To detect viruses, researchers have designed sensitive electrochemical biosensors [10,11] that use impedance change, before versus after viruses exposure, as the output signal [12]. However, the ultralow concentration of SARS-CoV-2 failed to be detected in real-time due to the non-amplified signals and limitations of the detection of the biosensors. Furthermore, for wearable masks that were designed with specific antibodies as the sensing element for real-time detection, the antibody receptors lost their bioactivity over time. To address this issue, researchers have recently integrated CRISPR-based biosensors with engineered circuits encoded in DNA or RNA, freeze-dried them, and allowed cell-free reactions to occur in the mask for the detection of SARS-CoV-2 [13]. However, this method still has limitations, such as the large detection errors caused by multiple components of the design, the loss of activity of the fluorescent probe over time, and the difficulty of precisely controlling the amplification factors.

Previously, a research group had embedded fibers and a collector [14] inside a wearable mask to collect viruses from breath for detection; however, it performed no better than when viruses were collected directly from a throat swab. The main challenges lie in the amplification of the signal output and the stability of detection. For wearable-mask biosensors, a sensitive and easy-to-test approach should collect the viruses to increase their concentration, amplify the signal output spontaneously, and monitor the viruses simultaneously.

Plasmonic resonance has emerged as a leading technique for the sensitive, robust, and facile detection of biomolecules [15] and includes Au/Ag/Cu/Al nanoparticles (NPs) [16]. The latter are extremely sensitive to the surface micro-environment and demonstrate large changes in their refractive index [17] and scattering intensity even for ultralow concentrations of biomolecules [18]. NP-based plasmonic sensors need precise control of the interparticle distance, size, and morphology of Au NPs because the resonance peak shift of NPs is extremely sensitive to interparticle distance, size, and morphology when detecting targets with refractive index changes. Resonance Rayleigh scattering (RRS) can overcome this problem by comparing the average electromagnetic field of RRS intensity in irregularly shaped Au nanoclusters (NCs). However, the stabilization of Au NCs [19] is crucial for long collection and self-detection sensors, which often agglomerate due to multiple factors, including their large surface area and large surface energy, leading to uncontrollable output signals and the loss of precision [20]. Improvement of the stability of Au NCs and the repeatability of the biosensor are critical issues to address to achieve highly sensitive detection over an extended period.

The unique bonding properties of Au NCs impact their size, ultimately influencing the intensity of the RRS observed from them. In this article, we propose an approach to achieve the ultrasensitive daily self-diagnosis of SARS-CoV-2 infection over an extended period via a flexible wearable mask. Firstly, heterogeneous nucleation was used to increase the stability of the Au NCs by pre-spurting titanium nanoclusters (Ti NCs) on the surface of quartz glass. This was achieved by decreasing the oxygen affinity and agglomeration of the Au NCs [21], as often done in their encapsulation for catalysis [22]. Thereafter, the previously reported SARS-CoV-2 spike protein N-terminal domain-binding aptamer 1 (SNAP1) [23] was immobilized on top of the Au NCs, instead of an antibody, as a target receptor to selectively capture SARS-CoV-2. This was inspired by a recent study that demonstrated aptamers immobilized with Au NCs remaining stable over 48 h even in vivo conditions [24]. Overall, the structure of Au NC-aptamers has great potential for the long-term and continuous collection and monitoring [25] of airborne viruses [24] in a facemask biosensor at room temperature. Considering the strict access to real viruses, the developed mask biosensor was validated using SARS-CoV-2 spike pseudovirus, and the liquefaction process to collect the viruses from breath was demonstrated via simulation. Finally, a machine learning approach, a support vector machine (SVM) classifier, was used to distinguish the RRS spectra from the same points before and after exposure to SARS-CoV-2 for up to 40 points. This significantly increased the accuracy of the measurements because it decreased errors caused by the instability of the Au NC -aptamer and Au NC-aptamer-virus. In conclusion, these biosensors showed stability in the long-term collection and self-detection of the pseudovirus and showed high potential for the reliable monitoring of viruses using specific aptameric closed-loop receptors.

## 2. Materials and Methods

### 2.1. Reagents and Materials

Artificial saliva (A7990) was purchased from Solarbio Life Science (Beijing, China). A freshly prepared, TE-buffered solution (10 mM Tri-HCl, 1 mM EDTA) was used throughout the study. A freshly prepared buffer B (4.5 g/L glucose, 0.1 g/L CaCl^2^, 0.2 g/L KCl, 0.2 g/L KH_2_PO^4^, 0.1 g/L MgCl^2^ 6H_2_O, 8 g/L NaCl, 2.1716 g/L Na_2_HPO^4^) was used for diluting BSA. Alcohol (99% wt) was purchased from Jiushan Chemical Ltd. (Nanjing, China). Tween-20, BSA, and TCEP (C4706-10G) were purchased from Sigma-Aldrich, PDMS (DC184), and quartz glasses were purchased from Suzhou Research Materials Microtech Ltd. (Suzhou, China). Glycerol and aptamers (Table 1) for SARS-CoV-2 were purchased from Sangon Biotech (Shanghai, China); SARS-CoV-2 pseudovirus (PSV-001) was purchased from Sino Biological (Beijing, China).

### 2.2. Equipment

Field emission scanning electron microscope (SEM) (Gemini 500, Zeiss, Cambus, UK), load-lock type sputtering system (CS-200z, Avatek Vacuum Technology Inc., Suzhou, China), power X-ray diffractometer (D8 advance, Bruker, Saarbrucken, Germany), dark field microscopy (Alpha300R, WItec, Ulm, Germany), environmental atomic force microscopy (AFM) (Cypher ES, Oxford instruments, Oxfordshire, England), X-ray photoelectron spectroscopy (XPS) (ESCALAB Xi+, Thermos fisher, Waltham, MA, USA), COMSOL (Version 5.5, COMSOL Inc., Stockholm, Sweden), and ANSYS fluent (Version 2021R1, ANSYS Inc., Canonsburg, PA, USA) were used for the corresponding tasks in this study.

### 2.3. Theory of the Biosensor

In the dark field, the incident light reached Au NCs, and the electromagnetic field of the incident light (Ei) plus the RRS electromagnetic field of Au NCs (Es) was equal to the obtained signal intensity (I_det_) at a given point. Es was the electromagnetic field of the Au NCs before and after binding, implying their contributions to the volume of the Au NC-aptamer and distribution of electrons as shown in Equations (1) [26]:

Idet = |Ei + Es|^2^ = |Ei|^2^ + |Es|^2^ + 2 Re (Ei Es*) (1)

where I_det_ is the obtained RRS light, Ei is the electromagnetic field corresponding to the dark field microscopy, and Es is the RRS electromagnetic field of the Au NC-aptamer.

Based on the Rayleigh scattering law, the theory of RRS intensity is expressed as follows by Equations (2) and (3) [26]:

(2) $$I_{\det}=\frac{8\pi^{4}r^{6}n_{med}^{4}}{\lambda^{4}d^{2}}\left(\frac{m^{2}-1}{m^{2}+2}\right)I_{0}(1+cos^{2}\theta )$$

(3) m=nnmed

where I_det_ is the RRS intensity at distance *d* from the Au NC-aptamer, *r* is the effective radius of Au NC-aptamer, $n_{med}$ is the refractive index of the microenvironment medium, and n is the refractive index of the Au NC-aptamer. d represents the distance between the Au NC-aptamer and the center of the objective lens, θ is the angle between obtained RRS light and incident light, and λ is the wavelength of the incident light.

### 2.4. Numerical Modeling of the Au Nanoclusters and Mask Biosensor

To characterize the change in color in the quartz glass with the deposition of Au NC layers, the scattering of Au NCs was simulated with commercial software COMSOL (Version 5.5). As can be seen, the scattering intensity changed with the interparticle distance. During human respiration cycles, computational fluid dynamic simulations were conducted with ANSYS Fluent software (Version 2021R1). It showed the fluid dynamic between the mask and the face.

### 2.5. Fabrication of the Biosensor

The quartz glass was washed with acetone, isopropyl alcohol, and de-ionized water step by step and ultrasonically cleaned for 5 min. Then, it was deposited on the Ti and Au using spurting, and the parameters of spurting were in Table S1.

### 2.6. Characterization of the Morphology of Nanoclusters Using AFM, SEM and XPS

Using environmental AFM, the surface morphology of Au NCs was characterized by different deposition times. Using field emission SEM, we characterized the surface morphology of the biosensor. The accelerating voltage (ETH) was 5 kV using an SE2 lens, and the working distance was 6 mm. XPS was used to show the binding energy of oxygen and Au NCs.

### 2.7. Functionalization of the Biosensor

After the Au NCs layer fabrication, this layer was functionalized with the previously proposed SARS-CoV-2 SNAP1, which was composed of pre-designed aptamers treated with 10 mM Tris(2-CarboxyEthyl) Phosphine (TCEP) in Tris-EDTA (TE) buffered solution. The TCEP can restore the oxidized sulfhydryl group since the Au NCs were easily oxidized when exposed to an oxygen-containing environment. TCEP is a reducing agent that was diluted with Tris hydrochloride (Tris HCl), which can keep the aptamers stable by binding them with the Au film via S-Au bonds. BSA (Bovine Serum Albumin) was diluted in a buffer B, and 1% BSA and saliva were injected into the microchannel step by step. Then, the biosensors were used to detect the pseudovirus.

### 2.8. Fitting the Resonance Rayleigh Scattering Spectra via Gaussian Function

Figure S1 (in Supplementary Material) exhibits the original scattering spectrum that was fitted based on the Gaussian function (equation and parameters given in Table 2). wi is the full width at half maximum of the peak of the RRS spectrum, and Ai is the area of the peak of the RRS spectrum. Table 3 shows a sample of fitting data. When the coefficient of determination was higher than 97%, the intensity of RRS was used in the comparison.

### 2.9. Analyzing Resonance Rayleigh Scattering Spectra via Support Vector Machine Classifier

The biosensor showed sensitivity, while the instability of the aptamers and physical absorption affected the performance of the biosensors. To reduce the errors of single-point measurement and decrease errors due to the instability of the Au NC-aptamer and Au NC-aptamer-virus, an SVM-based classifier was used to distinguish the spectra from the same points before and after exposure to SARS-CoV-2.

### 2.10. Statistics

We used the software ANSYS (Version 2021R1) and COMSOL (Version 5.5) for stimulation. Prism 7 was applied for data processing, and the scattering spectra were obtained using confocal dark field microscopy and then fitted using the Gaussian function.

## 3. Results

### 3.1. SARS-CoV-2 Detection in a Mask

**Figure 1:**
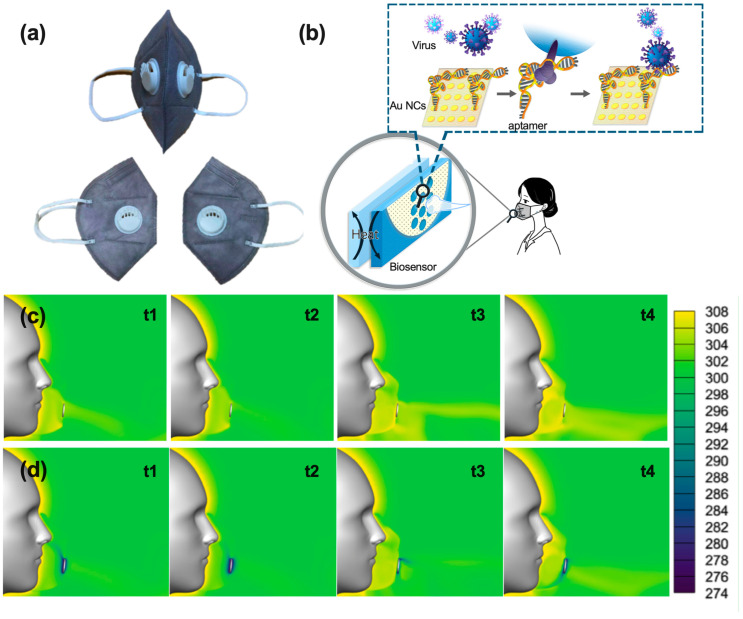
Design of the wearable mask for SARS-CoV-2 detection: (**a**) Mask containing two pockets for embedding biosensors, (**b**) process of liquefaction upon exhalation when breathing and the aptameric receptors catching SARS-CoV-2. Simulation results show temperature distributions around the human face and mask (**c**) at room temperature and (**d**) at 0 degrees (t1 and t3 is the middle time point of inhalation and exhalation, respectively, t2 and t4 is the time point between the inhalation and exhalation).

The design of the mask for the long-term collection and self-detection of SARS-CoV-2 from breath is shown in Figure 1a. The mask contained two pockets within fluid biosensors, which were embedded in a quartz layer with an ultrathin Au NC layer. When exhaling, the spray and steam of saliva from the breath would convert to liquid and form a mist on the surface of the ultrathin Ti NCs-Au NCs in low temperatures. The droplet, spray, and liquefaction liquid mixed and flowed on the surface of the Au NCs, and this mixture would then be collected automatically (Figure 1b). After packaging, the biosensors were embedded in the mask. The decorated aptamers on the Au NCs would capture the SARS-CoV-2 particles when exposed to them.

### 3.2. Investigation of the Mask Based Biosensor via ANSYS Simulation

To characterize the performance of the mask, we simulated the process using ANSYS Fluent software (Version 2021R1). The mask worked as a filter to collect SARS-CoV-2 from the mouth and nostrils in the inhalation and exhalation processes. The liquefaction process occurred in low temperatures. The temperature distributions in the vicinity of the human face and mask at room temperature and 0 degrees are shown in Figure 1c,d, respectively. The low-temperature region during the whole respiration cycle indicated the condensation of exhaled air. The biosensor layers collected the majority of SARS-CoV-2 released from the mouth and nostrils. After obtaining the RRS spectra and fitting them in the dark field, the RRS intensities of the spectra were compared for the same points before and after exposure to the targets.

### 3.3. Characterization of the Biosensor

**Figure 2:**
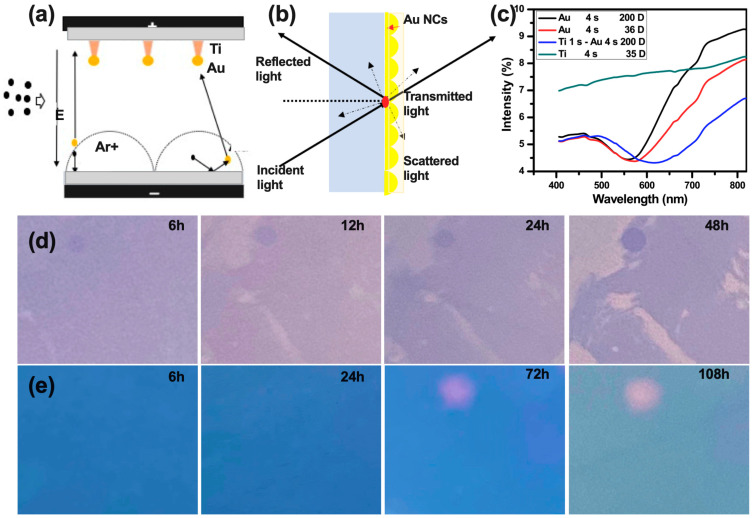
Characterization of the biosensor layer: (**a**) Mechanism of depositing Au NCs using spurting (E: Electric Field, Ar: Gas), (**b**) mechanism of detecting the transmittance, reflectance, and scattering light, (**c**) reflection spectra with different deposition with Ti and Au, (**d**) the color change in quartz glass after 4 s deposition of Au NCs over a period of 48 h, and (**e**) the color change in quartz glass after 1 s deposition of Ti NCs and 4 s deposition of Au NCs over a period of 108 h (E, electromagnetic field, D, degrees).

The heterogeneous nucleation for Au NCs, adapted in this work, was conducted by first depositing a layer of Ti NCs for 1 s via spurting on the quartz glass surface (Figure 2a). Gibbs’s free energy allowed the Au atoms to attach to the top of the pre-deposited Ti NCs, thereby forming an Au core on the surface of the quartz glass. Figure 2b shows the mechanism of the transmittance and reflectance on the surface of the quartz glass. The lower peak of the resonance absorption for the Au NCs increased significantly, compared with 4 s Ti NCs (Figure 2c) in reflection spectra from 400 to 820 nm wavelengths. In addition, temperature affected the resonance absorption since the full width at half maximum of the Au NCs at 200 degrees was smaller than at 0 degrees. And Au NCs with pre-deposition on 1 s Ti NCs showed a decreased resonance peak in the visible wavelength range. Figure 2d,e and Figure S2 show the photographs of the quartz glass for 4 s deposition of Au compared to that for 1 s deposition of Ti and 4 s deposition of Au, which indicated that the distance between the Au NCs changed, resulting in a change in reflection and scattered light. These results show that the color changed less when the Au NCs’ deposition followed the pre-deposition Ti, and the stability of bare Au NCs was weaker than that of the Ti pre-deposited ones. We then further investigated the stability of the Au NCs on the sensor surface after pre-deposition with Ti NCs. Figure S3 exhibits the binding energy of oxygen on the surface of the quartz glass using XPS with and without Ti NPs, while Figure S4 shows the binding energy of the Au NCs over 48 h, which was stable with time.

To examine the surface morphology of Ti NCs and Au NCs, SEM and AFM were utilized. SEM images were obtained to show the morphology of Ti NCs and Au NCs. Specifically, Figure S5 displays Ti NCs with a diameter of less than 3 nm after a deposition time of 1 s, while SEM images were used to compare the morphology of Au NCs following the initial 1 s deposition of Ti NCs and after an additional 4 s deposition of Au NC. The diameter of the irregularly shaped Au NCs was approximately 5 nm. Figure S6 exhibits the morphology of quartz glass after 1 s deposition of Ti NCs and then 4 s deposition of Au NCs using AFM. Furthermore, we simulated the scattering intensity of Au NCs, considering various distances in between the Au NCs using COMSOL software (version 5.5). As shown in Figure S7, the scattering intensity changed remarkably with increasing distance between the Au NCs.

### 3.4. Pre-Clinical Validation of Mask Based Biosensor Using the Artificial Spiked SARS-CoV-2 Pseudovirus Saliva Samples

For optical plasmonic biosensors, one of the key factors that affects the detection is achieving a stable optical path during the process of monitoring, which can minimize both background noise and detection errors caused by the unstable flow of the sample medium. The current work used dark field microscopy to collect the signals generated from the biosensors because of its lower background noise.

**Figure 3:**
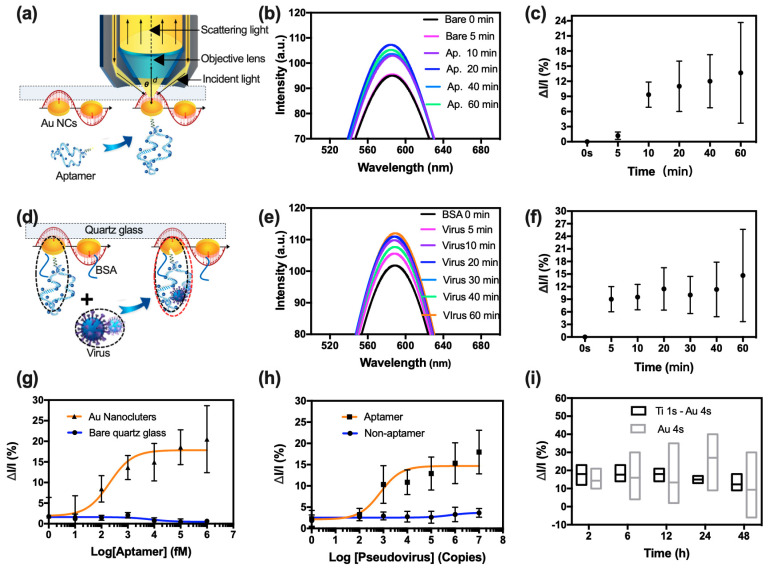
Validation of the wearable mask for SARS-CoV-2 detection: (**a**) Mechanism of Au NCs binding with aptamer. (**b**,**c**) change in RRS spectra and intensity when decorating Au NCs with 0.1 nM aptamers versus time, (**d**) mechanism of Au NC-aptamer capturing pseudovirus, the effective radium of Au NC-aptamer change from black dot line to the red dot line on the surface of quartz glass, (**e**,**f**) change in RRS spectra and intensity when capturing pseudovirus of 10^5^ at fixed 0.1 nM aptamers versus time, (**g**) change in RRS intensity of Au NCs when binding with aptamers range from 1 fM to 1 nM (orange line) versus bare quartz glass (blue line), (**h**) change in RRS intensity when capturing with different concentrations of pseudovirus at a fixed aptamer concentration of 0.1 nM (orange line) versus non-aptamer (blue line), (**i**) change in RRS intensity change in 4 s of Au only and in 1 s of Ti and 4 s of Au NCs with time. Error bars are ± SD with N = 3 samples per group (**c**,**f**–**i**) (ap, aptamer; RRS, resonance Rayleigh scattering; NCs, nanoclusters).

Figure 3a shows the optical path of the biosensor in this study, the aptamer bound directly to Au through the sulfhydryl group(S). The incident light went through the transparent quartz glass and reached the Au NC-aptamer, the *d* between the objective lens and Au NCs was 9 ± 0.005 mm, the θ between them received RRS light, and the incident light was 50.7 degrees. The RRS intensity changed significantly with surface microenvironment changes. Figure 3b shows the fitting spectra when decorating Au NCs with 0.1 nM aptamers versus time, and Figure 3c showed that the RRS intensity changed with time after fitting. The same fitting method was used in the following experiments.

For the special capturing of SARS-CoV-2 with the pre-decorating aptamers, the biosensor surface was treated with 1% BSA to minimize non-specific binding, and the medium input was set at 100 uL per min. It was decorated with aptamers at a concentration of 0.1 nM, and the functionalized Au NCs were used to detect the pseudovirus in saliva. The RRS intensity changed after exposure to the pseudovirus. Figure 3d shows the change in the nanostructure and effective radius of Au NC-aptamer, with the black and red dot ellipses labeling the effective radius of the Au NC-aptamer both before and after capturing the pseudovirus. Figure 3e,f show the RRS spectrum and intensity change after exposing the pseudovirus with time. The RRS intensity exhibited changes upon exposure to the pseudovirus, while the errors increased over time. Figure 3g displays the RRS intensity at varying concentrations of aptamers ranging from 0.1 pM to 1 nM. Additionally, Figure 3h shows the RRS intensity in capturing pseudoviruses ranging from 10^3^ to 10^7^ copies at a fixed concentration of 0.1 nM for the aptamer. Notably, the two blue lines representing the bare quartz glass and non-aptamer biosensor showed no increase in RRS intensity with changes in the aptamer and pseudovirus concentration. In comparison, the increasing ratio of RRS intensity was approximately 10% at 0.1 pM and reached 30% at 1 nM, and the increasing ratio of RRS intensity was approximately 10% when exposing 10^5^ copies of the pseudovirus. Furthermore, a GraphPad software (Version 7.0a) model was used to fit these changes in RRS intensity with the pseudovirus concentration by the following Equation (4):

(4) $$\Delta I=14.69-\frac{12.562}{1+(10^{C\left(pseudovirus\right)-2.869})}$$

The limit of detection is approximately 10^3^ copies with a 3% increase. Figure 3i illustrates a comparison between the RRS intensities of bare Au NCs and Au NCs with pre-deposited Ti NCs, both binding with the aptamer at a fixed concentration of 0.1 nM and capturing the pseudovirus at a concentration of 10^5^ copies. The results indicated that the Au NC-aptamer with pre-deposited Ti NCs exhibited a greater sensitivity and stability than the bare Au NCs.

### 3.5. Investigation of the Resonance Rayleigh Scattering Spectra via Support Vector Machine Classifier

**Figure 4:**
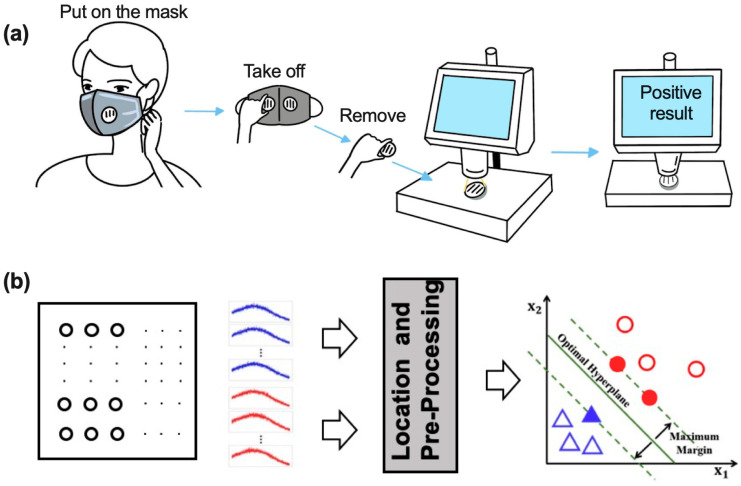
Process of using the mask and analyzing the data: (**a**) Process of using the mask and detecting SARS-CoV-2, (**b**) a machine learning support vector machine classifier was introduced to compare the spectra before (blue) and after (red) exposure to SARS-CoV-2.

Figure 4a shows the process of wearer operation to analyze the SARS-CoV-2 detection results. As can be seen, the wearer took off the biosensor from the mask and then put it on a monitoring device to detect SARS-CoV-2, which would show positive/negative results. Figure 4b is the classification process. More than 40 points from the surface of the quartz glass were randomly selected and compared with the RRS intensity, which was labeled from 1 to 40. The blue and red curves were RRS spectra from the same point before and after exposure to the pseudovirus. The method showed that at least 83% of the points displayed similar results, which increased the RRS intensity when the biosensors were exposed to SARS-CoV-2.

## 4. Discussion

To reduce the impact of COVID-19, early detection and monitoring methods are being continuously developed. In this study, we introduced a novel and stable technique that utilizes heterogenous-nucleation Au NCs through the pre-deposition of Ti NCs on the surface of quartz glass for the long-term collection and self-detection of SARS-CoV-2. For comparison, several biosensor prototypes for detecting SARS-CoV-2 in masks are shown in Table 4. This work shows easy fabrication and stabilization with an external detector, and the sensor is sensitive to long-term collection to amplify the concentration of a pseudovirus.

Simultaneously, while the mask biosensors show promising results, there are still some limitations that need to be addressed. For instance, it is crucial to control the physical absorption of air effectively. Even the pre-deposition of Ti NCs can increase the stabilization of Au NCs, which resulted in them being highly stable for up to 48 h; the biosensor shows sensitivity for up to 1 h, butt falls short of the desired 48 h lifespan due to the instability of the aptamers and physical absorption. Therefore, it is imperative to design and upgrade the biosensor surface to enhance the bioactivity and stability of the aptamers. Additionally, the current study did not utilize real human samples to demonstrate the biosensor’s efficacy in real-world scenarios. These biosensors have only tested the pseudovirus in saliva with a controllable microenvironment. To improve upon these limitations, future research can be directed toward addressing these issues.

## 5. Conclusions

In this article, the RRS intensity of the Au NC-aptamer complex was used as a signal for monitoring the virus. The average of the electromagnetic field of the Au NC-aptamer complex can overcome the precise control of NPs and the resonance peak shift of NPs in monitoring targets. Further, compared to the bare Au NCs, which were stable for less than 6 h, the heterogeneous nucleation of Ti NCs and Au NCs remained stable for up to 48 h because of the adhesion of Ti NCs. The biosensor based on Au NC-aptamers was sensitive for up to 1 h, and the increase in errors over time can be attributed to the instability of both the Au NC-aptamer and Au NC-aptamer-virus complexes. The single detection showed large background noises with the limit detection of 10^3^ copies of the pseudovirus in saliva. Using an SVM classifier, a high number of points was concurrently detected from a single biosensor and the RRS intensity changes were distinguished. This significantly increased the accuracy of the measurements because it decreased errors due to the instability of the Au NC-aptamer and Au NC-aptamer-virus. Our method was easy to use and had a detection limit lower than 10^3^ copies of the pseudovirus in saliva. It showed potential for becoming a reliable measurement tool. The novel approach was both time-saving and cost-effective, offering a promising solution for the early monitoring of SARS-CoV-2, including its latest variants as well as other infectious diseases.

## Figures and Tables

**Table 1:** Spike protein N-terminal domain-binding aptamers parameters.

DNA Sequence	TCGCTCTTTCCGCTTCTTCGCGGTCATGTCATCCTGACTGACCCTAAGGTGCGAACATCGCCCGCGTAAGTCCGTGTGTGCGAA
OD	5.0
Purification	HPLC
Modification	5′SH C6
Modification molecular weight	196.20
Primer length	86
Molecular weight	26,361.03
Aggregate molecular weight	26,557.23

OD, optical density; HPLC, high-performance liquid chromatography.

**Table 2:** Functions and parameters for fitting the spectra.

Description	
$y=yo+\overset{n-1}{\underset{i=0}{\sum }}Ampi\;e^{-0.5{{(}^{\frac{x-xi}{si}})}^{2}}$	
$wi=si\sqrt{8\ln\left(2\right)}$	
$Ai=Ampi\;si\;\sqrt{2\pi }$	
n	Number of Gaussian functions
y	Offset
xi	Center
si	Width (standard deviation)
Ampi	Amplitude
Wi	Width (FWHM)
Ai	Area

FWHM, full width at half maximum.

**Table 3:** Fitting spectra parameters.

Description	
No. of sample points	5758.00
Degrees of freedom	5748.00
CoD (R^2^)	0.98
Correlation	0.99
Average error	14.65

CoD (R^2^), coefficient of determination.

**Table 4:** Comparison of the general performance of the literature-reported wearable-mask biosensors for SARS-CoV-2 detection (biosensors for the mask are prototypes).

Sl. No.	Technologies	Drawbacks	Advantages	Signal Amplification	Limit of Detection	Reference
1	Impedance changes	No collector to increase the signals	Easy operation	No	7 pfu/mL (aerosols)	[12]
2	Anti-FITC conjugated Au NPs	Uncontrol reaction and instability	Easy operation	Yes	500 copies	[13]
3	Wearable collector	No biosensor and external detection	Sensitivity	No	10,000 copies	[14]
4	Resonance Rayleigh Scattering intensity	External detector	Easy fabrication Sensitivity Stabilization	Yes	1000 copies	This work

FITC, fluorescein isothiocyanate isomer; NPs, nanoparticles.

## Data Availability

Not applicable.

## Supplementary Materials

The following supporting information can be downloaded at: https://www.mdpi.com/article/10.3390/bios13090858/s1, Table S1: Parameters of the deposition of Au NCs. Figure S1: Resonance Rayleigh scattering spectrum and its fitting curve. Figure S2: color changes of the quartz lass after deposition of Au nanoclusters at 48 h and 108 h; Figure S3: Binding energy of oxygen. Figure S4: The full width at half maximum of bonding energy of Au nanoclusters over time; Figure S5: Morphology of the Ti nanoclusters and Au nanoclusters using the SEM, (a) Ti nanoclusters, (b) Au nanoclusters; Figure S6: Morphology of the Au nanoclusters using the AFM; Figure S7: The scattered light changes with the distance of Au nanoclusters, and the diameter of the nanoclusters is 10 nm.
