# Supplementary material for: Heterogeneous-Nucleation Biosensor for Long-Term Collection and Mask-Based Self-Detection of SARS-CoV-2
Source: Biosensors (Basel). 2023 Aug 30;13(9):858. doi: 10.3390/bios13090858 (PMC10526364; doi:10.3390/bios13090858)
Supplement: Supplementary file 1 [file biosensors-13-00858-s001.zip › biosensors-2534377-supplementary.pdf]

Supplementary Materials

Heterogeneous-Nucleation Biosensor for Long-Term Collection and Mask-Based Self-Detection of SARS-CoV-2

Yi Su, Sumin Bian, Dingyi Pan, Yankun Xu, Guoguang Rong, Hongyong Zhang and Mohamad Sawan

Table S1. Parameters of the deposition of Au nanoclusters.

**Parameters**	
**Power(W)**	200
Time(s)	2 / 4 /8 /15
Distance between wafer and material (cm)	10
Rotation (r/s)	13
Temperature (℃)	200

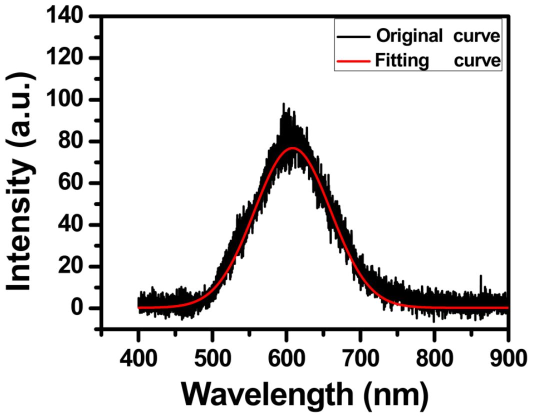

Figure S1. Resonance Rayleigh scattering spectrum before and after fitting.

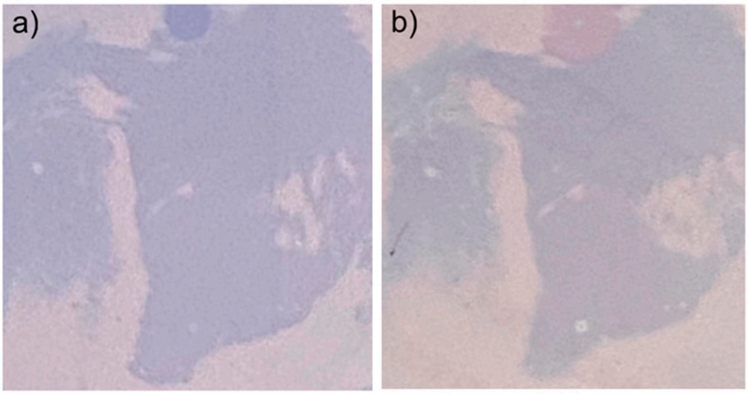

Figure S2. Color changes of the quartz glass after deposition Au nanoclusters at 48 h (a) and 108 h (b).

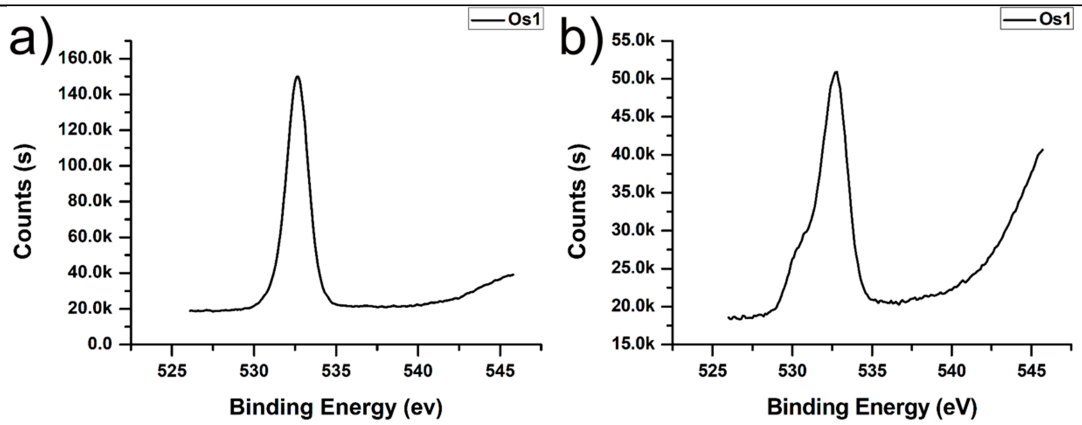

Figure S3. Binding energy of oxygen. a) Binding energy of oxygen on the surface of quartz glass with Au nanoclusters and without Ti nanoclusters, and b) Binding energy of oxygen on the surface of quartz glass with 1 s deposition of Ti nanoclusters and 4 s deposition of Au nanoclusters (Os1: state of oxygen).

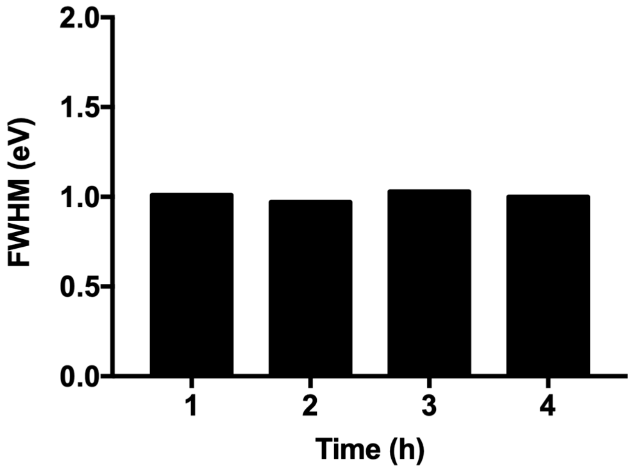

Figure S4. The full width at half maximum of bonding energy of Au nanoclusters over time (FWHM: full width at half maximum).

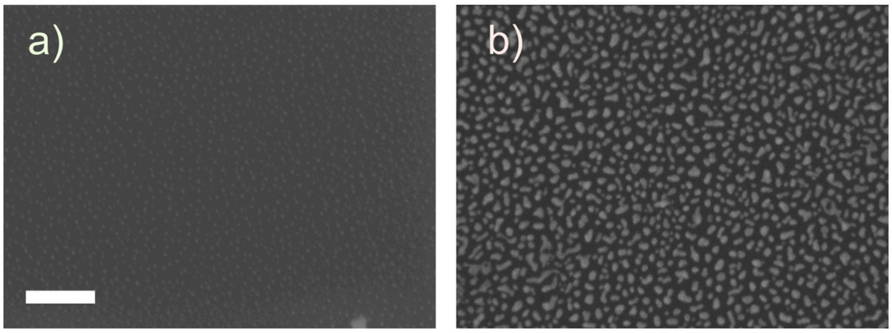

Figure S5. Morphology of the Ti nanoclusters and Au nanoclusters using the SEM, a) Ti nanoclusters, b) Au nanoclusters (Scale bar :50 nm). .

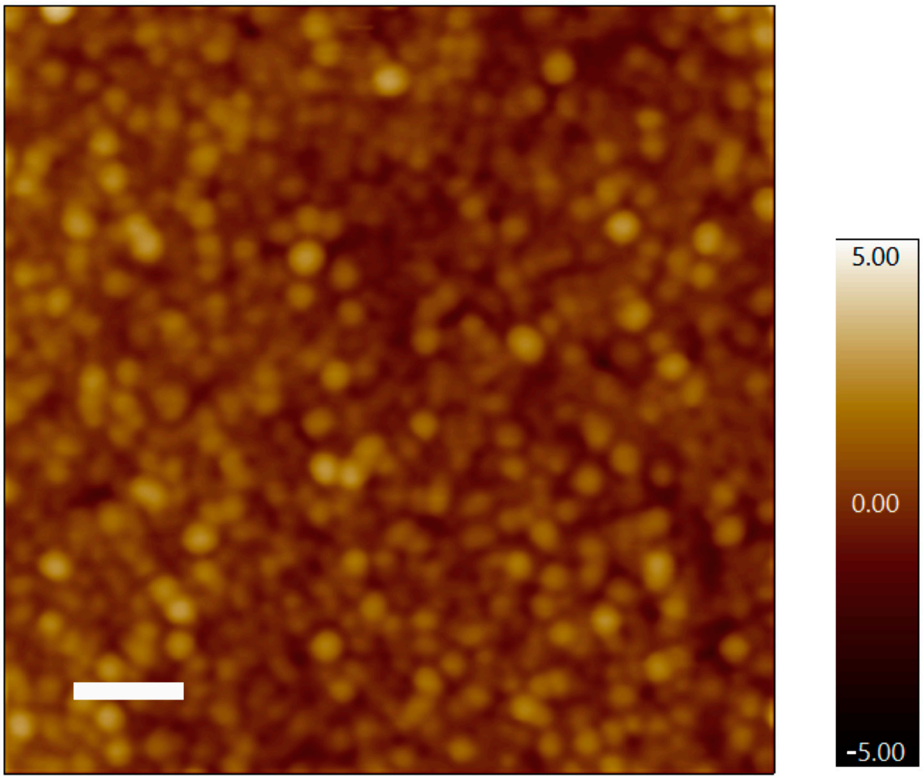

Figure S6. Morphology of the Au nanoclusters using the AFM (Scale bar :50 nm).

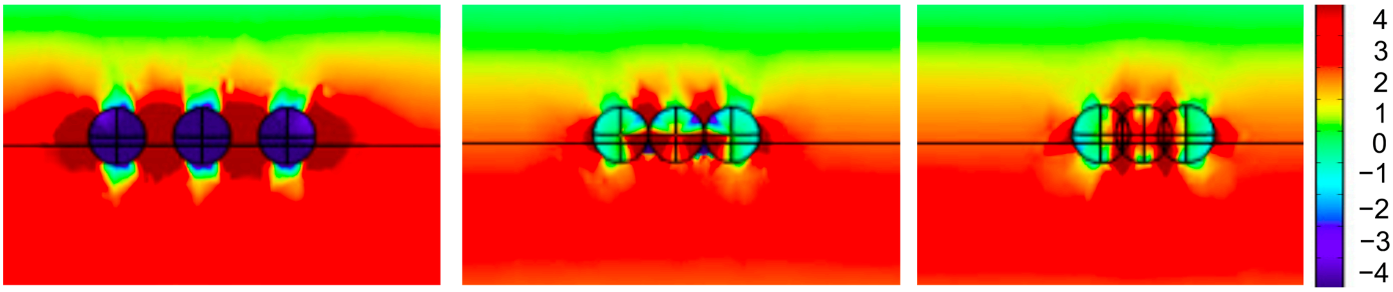

Figure S7. The scattered light changes with the distance of Au nanoclusters, and the diameter of the nanoclusters is 10 nm.
